# Prediction of piRNAs using transposon interaction and a support vector machine

**DOI:** 10.1186/s12859-014-0419-6

**Published:** 2014-12-30

**Authors:** Kai Wang, Chun Liang, Jinding Liu, Huamei Xiao, Shuiqing Huang, Jianhua Xu, Fei Li

**Affiliations:** Department of Entomology, College of Plant Protection, Nanjing Agricultural University, Nanjing, 210095 China; Department of Biology, Miami University, Oxford, OH 45056 USA; Department of Computer Science and software Engineering, Miami University, Oxford, OH 45056 USA; College of Information and Technology, Nanjing Agricultural University, Nanjing, 210095 China; College of computer science and Technology, Nanjing Normal University, Nanjing, 210023 China; Currently affiliation: Department of Biology, Miami University, Oxford, OH 45056 USA

**Keywords:** piRNAs, piRNA prediction, Support vector machine (SVM), *Chilo suppressalis*, *Drosophila melanogast*er, *Homo sapiens*, *Mus musculus*, *Rattus norvegicus*

## Abstract

**Background:**

Piwi-interacting RNAs (piRNAs) are a class of small non-coding RNA primarily expressed in germ cells that can silence transposons at the post-transcriptional level. Accurate prediction of piRNAs remains a significant challenge.

**Results:**

We developed a program for piRNA annotation (Piano) using piRNA-transposon interaction information. We downloaded 13,848 *Drosophila* piRNAs and 261,500 *Drosophila* transposons. The piRNAs were aligned to transposons with a maximum of three mismatches. Then, piRNA-transposon interactions were predicted by RNAplex. Triplet elements combining structure and sequence information were extracted from piRNA-transposon matching/pairing duplexes. A support vector machine (SVM) was used on these triplet elements to classify real and pseudo piRNAs, achieving 95.3 ± 0.33% accuracy and 96.0 ± 0.5% sensitivity. The SVM classifier can be used to correctly predict human, mouse and rat piRNAs, with overall accuracy of 90.6%. We used Piano to predict piRNAs for the rice stem borer, *Chilo suppressalis*, an important rice insect pest that causes huge yield loss. As a result, 82,639 piRNAs were predicted in *C. suppressalis*.

**Conclusions:**

Piano demonstrates excellent piRNA prediction performance by using both structure and sequence features of transposon-piRNAs interactions. Piano is freely available to the academic community at http://ento.njau.edu.cn/Piano.html.

**Electronic supplementary material:**

The online version of this article (doi:10.1186/s12859-014-0419-6) contains supplementary material, which is available to authorized users.

## Background

Non-coding RNAs (ncRNAs) are important RNA molecules. Although they do not encode proteins, their roles in gene regulation are crucial [1,2]. There are many types of long ncRNAs whose functions remain largely unknown [3]. Short ncRNAs, such as microRNAs (miRNAs) and piwi-interacting RNAs (piRNAs), are important post-transcriptional regulators [4]. piRNAs are produced from un-characterized precursors in both male and female germline cells. The discovery of piRNAs was a highly important break-through as they are involved in germ cell formation, germline stem cell maintenance, spermatogenesis and oogenesis [5-8].

The biogenesis of piRNAs is quite different from that of miRNAs. Although details of their biogenesis are currently unclear, several models have been proposed. In germline cells, piRNAs can be produced by the primary processing pathway and by a feed-forward loop, called the “ping-pong” pathway, which uses primary piRNAs to direct cleavage of complementary transposon sense transcripts [9]. These mature sense piRNAs will target complementary antisense piRNA precursors to create mature antisense piRNAs that can continue sense piRNA generation. piRNAs lack apparent structural motif and sequence conservation across different species, making their prediction a difficult task. piRNAs are generally understood to participate in transposon silencing during embryo development [10]. The majority of piRNAs are antisense to transposons. In the genome, piRNAs tend to occur in clusters and to be located in intergenic regions [5]. However, piRNAs are also found in somatic cells [11], and studying piRNA functionality is still a challenging task because of the wide variation of piRNA sequences.

piRNAs have been reported in human [12], mouse [6], rat [13], zebra fish [7], and fruit fly [14]. A typical experimental procedure to obtain piRNA data relies on immunoprecipitation of small RNAs bound to the protein PIWI and deep sequencing. However, with this method, it is still hard to identify piRNAs expressed at low levels or with restricted spatiotemporal expression. Therefore, computational prediction can provide an alternative approach to identify potential piRNAs. Unfortunately, homology sequence searching methods such as BLAST [15] or motif searching methods such as MEME [16] are not suitable for detecting piRNAs because sequence conservation is very low and no conserved structural motif has been detected in piRNAs.

The first *de novo* algorithm to identify piRNAs was a position-specific usage method that classifies piRNA sites along the genome using piRNAs starting with a uridine at their 5′ ends. A vector of 21 × 4 components was constructed containing 10 nucleotides upstream and 10 downstream of the starting U (i.e., +10 to −10, where U has the position of 0). The precision of this algorithm was only 61-72%, indicating that this tool is helpful for piRNA classification but still needs improvement [17]. Zhang *et al.* developed a *k*-mer based algorithm, named piRNApredictor, to predict piRNAs. piRNA and non-piRNA sequences from five model species were used as the training set. piRNApredictor has a high precision of >90% and a sensitivity of >60% [18]. piRNApredictor was integrated with mirTools 2.0 to predict piRNAs from small RNA-Seq data [19]. Moreover, iMir can be used to find piRNAs [20], but it mainly focuses on miRNAs. There is another program called "multiclass relevance units machine" that shows an excellent performance on piRNA classification [21]. However, it focuses on algorithm development and its software is not publicly available. proTRAC [22] and piClust [23] were developed to display known piRNA clusters, but they cannot be used to find new piRNAs.

Here, we present a new program, piRNA annotation (Piano), to predict piRNAs using piRNA-transposon interaction information. A support vector machine (SVM) was used to classify real piRNAs and pseudo piRNAs. Our analysis of *Drosophila melanogaster* data shows that Piano performs well in piRNA prediction, with over 90% prediction sensitivity, specificity and accuracy. The SVM classifier trained with *Drosophila* piRNA data can also accurately identify piRNAs of other species such as *Homo sapiens*, *Mus musculus* and *Rattus norvegicus*. Using small RNA-Seq data, Piano was successfully used to predict piRNAs for an important rice pest, the rice striped stem borer, *Chilo suppressalis*.

## Methods

### Training and testing sets

Two datasets were built for *D. melanogaster*: one contained real piRNAs and the other contained pseudo piRNAs. We downloaded 987 piRNAs from the NCBI GenBank database (GI: 157361675–157362817) [24] and 12,903 piRNAs from the NCBI Gene Expression Omnibus with the accession number GSE9138 [14]. By using short sequence alignment software, SeqMap [25], highly similar sequences were removed. After removing redundancy, 13,848 non-redundant piRNAs were kept. We downloaded 261,500 *Drosophila* transposons from the UCSC Genome Browser (Apr. 2006 dm3) [26]. We aligned 13,848 piRNAs to the transposon sequences using SeqMap with a maximum of three mismatches allowed. Among 13,848 non-redundant piRNAs, 9,758 (70.4%) could be aligned successfully, suggesting that they can target transposons.

Since DNA sequences are not random sequences, there are some differences between coding and non-coding RNAs. Because piRNAs are non-coding RNAs, we used non-coding RNAs as a negative control to generate our pseudo piRNA dataset. We downloaded 102,655 *Drosophila* ncRNA sequences from the NONCODE v3.0 database [27]. First, we removed all piRNAs from this dataset. We then randomly selected one ncRNA sequence and randomly cut out a short sequence of 20–30 nt as one candidate sequence. By this double-randomization process, we were able to obtain about 200,000 candidate pseudo piRNAs. Next, we mapped all these candidate sequences to the transposons with a maximum of three mismatches, and those sequences that did not map to the transposons were removed from the candidate sequence dataset. Accordingly, we produced 38,919 non-redundant candidate pseudo piRNAs. We then randomly selected some candidate pseudo piRNA sequences to simulate the length distribution of real piRNAs. Finally, we obtained 9,240 sequences that formed the pseudo piRNA dataset as the negative dataset for SVM classification.

### Cross-species test set

We applied the SVM classifier trained with *Drosophila* piRNAs to human, mouse and rat data. In total, 32,152 human, 75,814 mouse and 66,758 rat piRNAs were downloaded from the NONCODE v3.0 database [27]. Transposons of the three species were downloaded from the UCSC Genome Browser [26], including 8,537,572 human, 7,320,714 mouse and 6,380,192 rat transposons.

### Structure-sequence triplet elements

**Figure 1:**
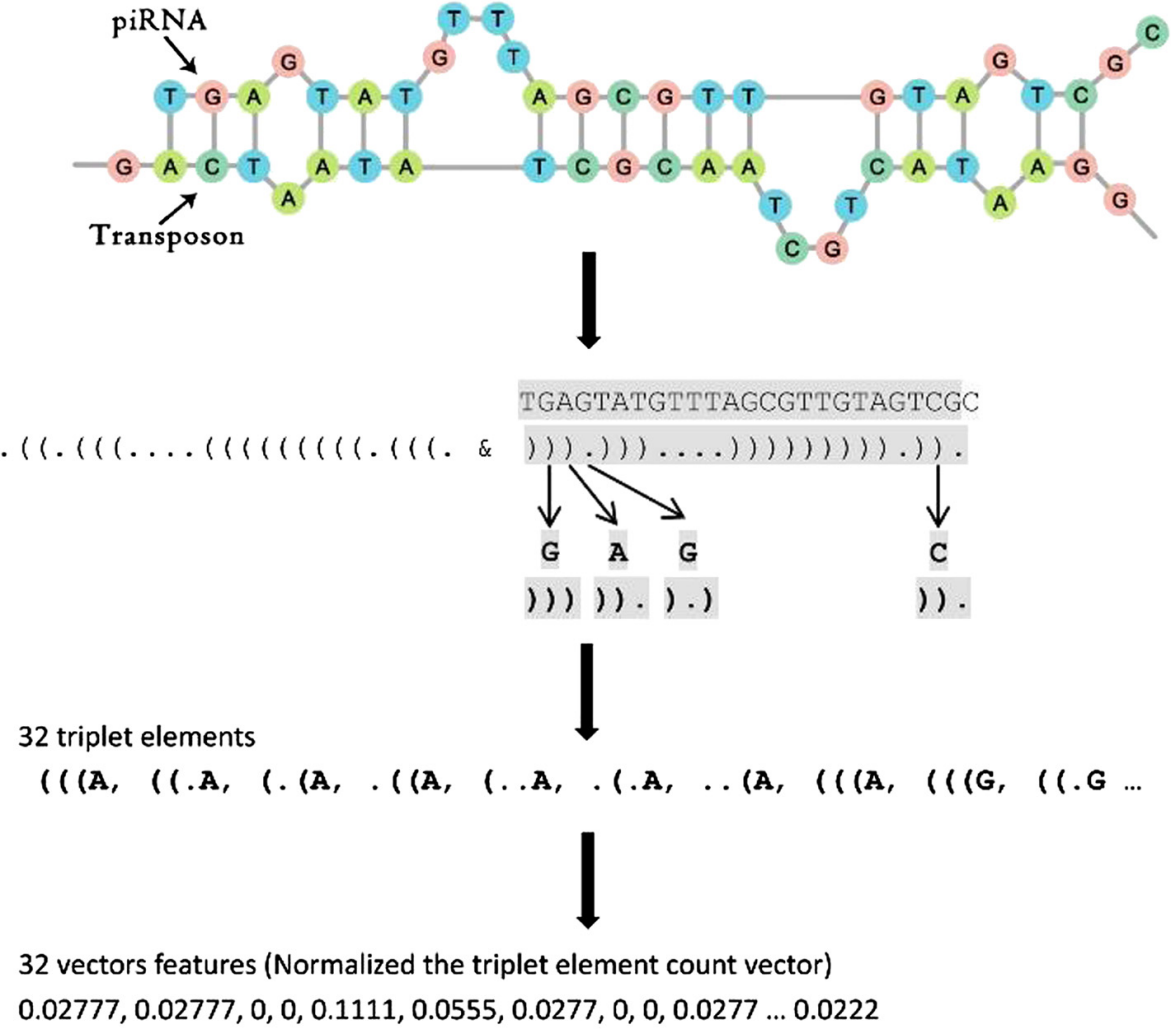
**Principle of triplet elements.**

The main function of piRNAs is to silence transposons. To target transposons, piRNAs need to bind with their target sequences. In piRNApredictor [18], 1,364 *k*-mer strings (k = 1, 2, 3, 4, 5) were used to describe piRNA sequences. Although this *k*-mer approach is a good way to characterize and extract sequence content features from piRNAs, it is purely a mathematical method that might lack biological insight and significance. In our program, we analyzed piRNA-transposon interaction information using RNAplex [28]. When a piRNA binds with a transposon, there are two statuses for each nucleotide of the piRNA, paired or unpaired (see Figure 1). The paired nucleotides of piRNAs are indicated by opening brackets "(" whereas the paired nucleotides of transposons are indicated by closing brackets ")". The unpaired nucleotides in both piRNAs and transposons are indicated by dots “.”. For any three adjacent nucleotides of a piRNA, there are 8 (2^3^) possible structure compositions: "(((", "((.", "(..", "(.(", ".((", ".(.", "..(" and "…". Combining the middle nucleotide of each three adjacent nucleotides, we can form 32 (4 × 8) different triplet elements that contain both structural information of transposon-piRNA alignment/pairing and piRNA sequence information, which we call structure-sequence triplet elements (see Figure 1). For instance, the triplet element “(((U” indicates that three contiguous piRNA nucleotides are aligned with a transposon and the middle nucleotide is U.

### Support vector machine

Support vector machines (SVMs) have been widely applied in the classification of biological signals. For a given dataset, *x*_*i*_ ∈ *R*_*n*_ (*i* = 1,…*N*) with corresponding labels *y*_*i*_ (*y*_*i*_ = +1 or −1, representing real and pseudo piRNAs respectively in this work), SVM gives a decision function ${\displaystyle \int }(x)=sgn\ \left({\displaystyle {\sum}_{i=1}^N}{y}_i{\alpha}_iK\left(x,{x}_i\right)+b\right)$, where *α*_*i*_ represents the coefficients to be learnt and K is the kernel function. The LibSVM3.12 package (http://www.csie.ntu.edu.tw/~cjlin/libsvm/) [29] was used to perform the analysis. For optimizing the SVM classifier, the penalty parameter *C* and the RBF kernel parameter *γ* were adjusted using the grid search strategy in LibSVM.

### Prediction system assessment

Prediction accuracy (ACC), specificity (*Sp*), precision (*Pre*) and sensitivity (*Se*) are widely used to evaluate the algorithm performance. The equations for these parameters are given below, with the following abbreviations: false positive (FP), true positive (TP), false negative (FN) and true negative (TN).

$$ Se=\frac{TP}{TP+FN}\times \kern0.5em 100\% $$

$$ Sp=\frac{TN}{TN+FP}\times \kern0.5em 100\% $$

$$ Pre=\frac{TP}{TP+FP}\times \kern0.5em 100\% $$

$$ \mathrm{A}\mathrm{C}\mathrm{C}=\frac{TP+TN}{TP+TN+FP+FN}\times \kern0.5em 100\% $$

## Results and discussion

### SVM classification

We used a SVM to classify real and pseudo piRNAs using 32-dimensional vectors of structure-sequence triplet elements. The training dataset was randomly divided into ten equally sized partitions. Each partition had the same ratio of positive samples to negative samples. Seven partitions were merged together as the training dataset. Two of the other partitions were merged together to validate the classifier for model selection. The tenth partition was used as the testing dataset. We used 10-fold crossing validation to improve the reliability. The training procedure was repeated ten times with different combinations of training set (seven partitions), validation set (two partitions) and testing set (one partition) (Table 1). We called our program that uses a SVM classifier with structure-sequence triplet elements to predict piRNAs, the piRNA annotation platform, abbreviated as Piano.

**Table 1:** **Datasets for SVM classification**

	**Positive samples**	**Negative samples**
Training set	6,833	6,468
Validation set	1,950	1,848
Test set	975	924
Total number	9,758	9,240

**Figure 2:**
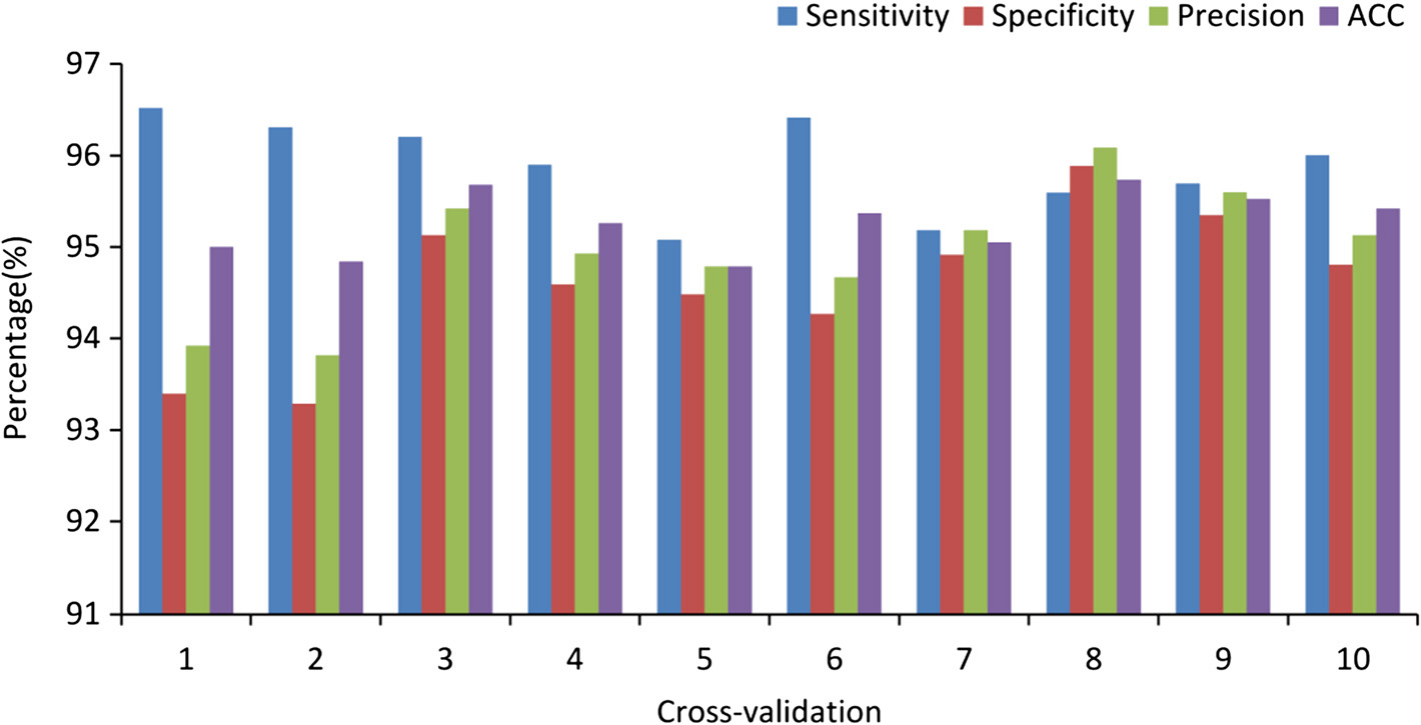
**10-cross-validation results.**

In one of these tests, Piano correctly recognized 935 out of 975 real *Drosophila* piRNAs, and detected 874 out of 924 pseudo piRNAs as negative cases (Additional file 1: Table S2). We calculated the average value of ten tests. Piano gives a sensitivity of 95.89 ± 0.50%, specificity of 94.61 ± 0.81%, accuracy of 95.27 ± 0.34%, and precision of 94.95 ± 0.71% (Figure 2). The high performance of Piano indicated that real and pseudo piRNAs are quite different in terms of structure-sequence triplet elements. The triplet elements combine both structural information of piRNA-transposon alignment/pairing and sequence information of the middle nucleotide of three contiguous piRNA nucleotides. Such a structure-sequence triplet element was previously used to classify real and pseudo miRNAs [30], suggesting that this structure-sequence feature might be common for small ncRNAs. Although pseudo piRNAs are also antisense to transposons due to their alignment (see Methods), they can be effectively distinguished from real piRNAs by the triplet elements, demonstrating that piRNA-transposon interaction information is an intrinsic characteristic of piRNAs.

**Figure 3:**
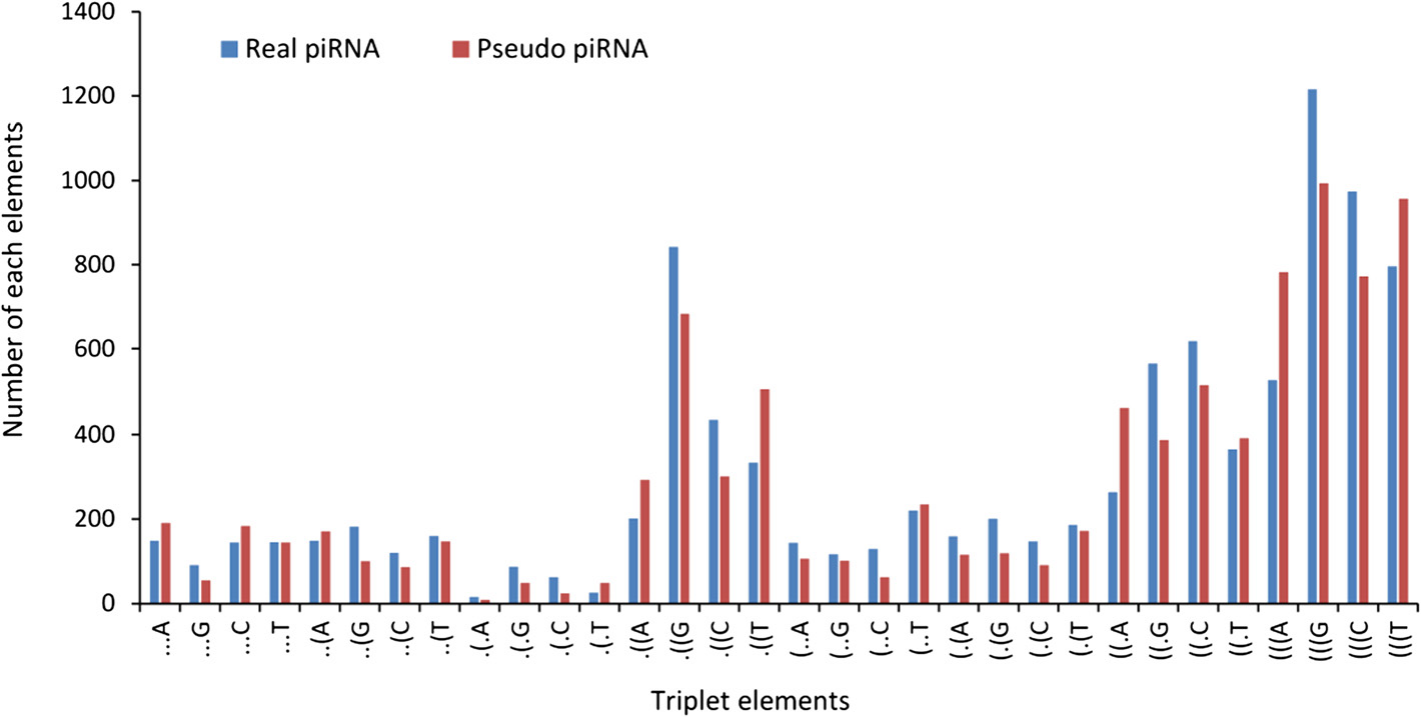
**The distribution of triplet elements in two datasets (pseudo piRNA vs. real piRNA).**

We calculated the average frequencies of the 32 structure-sequence triplet elements in the real piRNAs and pseudo piRNAs. Our data analysis indicated that "(((G" and "(((C" appear at higher frequencies in real piRNAs than in pseudo piRNAs. The group of two-paired nucleotides and one unpaired (e.g., "((.A") appears more often in pseudo piRNAs than in real piRNAs (Figure 3). We calculated the F-value to estimate the discriminative power of the different triplet elements [31,32].

$$ F\left({x}_j\right)=\left|\frac{\mu_j^{+}-{\mu}_j^{-}}{\sigma_j^{+}+{\sigma}_j^{-}}\right| $$

For each feature *x*_*j*_, *j* = 1, …, *N*, we calculated the mean ${\mu}_j^{+}$ (${\mu}_j^{-}$) and standard deviation ${\sigma}_j^{+}$ (${\sigma}_j^{-}$) using positive or negative examples, respectively. The results demonstrated that “…G”, “(.(G”, “..(C”, “..(G”, and “(..C” are the top five discriminative elements. Four of them contain continuously unpaired nucleotides, suggesting that binding stability between piRNA-transposon interactions is the key information in classifying real and pseudo piRNAs (Additional file 2: Table S1).

### Application of Piano to other species

To test the robustness of the program, we used the SVM classifier trained using the aforementioned *Drosophila* piRNA dataset to predict human, mouse and rat piRNAs. After aligning 32,152 human, 75,814 mouse and 66,758 rat piRNAs to relevant transposon sequences, 7,140 human, 14,495 mouse and 14,195 rat piRNAs were alignable and used in our cross-species application. The SVM classifier correctly recognized 6,690 out of 7,140 human (93.7%), 12,915 out of 14,495 mouse (89.1%) and 12,737 out of 14,195 rat piRNAs (89.7%). This gives an overall accuracy of 90.9% for the three cross-species datasets (Table 2).

**Table 2:** **Cross-species validation results**

**Test set**	**Size**	**ACC (%)**
*H. sapiens*	7,140	93.7
*M. musculus*	14,495	89.1
*R. norvegicus*	14,195	89.7

The high accuracy in predicting mammalian piRNAs achieved by the SVM classifier trained with *Drosophila* piRNAs suggests that the structure-sequence triplet element represents a conserved feature for piRNAs.

### Comparison with other methods

Piano was compared with piRNApredictor, which was developed by Zhang *et al.* (2011). We used the same datasets to test the performance of these two methods. For each species, the testing data were composed of real piRNAs and pseudo piRNAs, all of which were mapped to the relevant transposon sequences (mismatch < =3). When predicting mouse piRNAs, compared with the algorithm proposed by Betel *et al.* (2007), piRNApredictor had high precision, 95.53%, and the sensitivity was 72.47% with the default parameter (t = 2). This means that piRNApredictor is good at recognizing positive but not negative samples. When comparing Piano and piRNApredictor with our datasets, Piano achieves higher sensitivity, specificity and accuracy than piRNApredictor (Table 3).

**Table 3:** **Comparison between results from Piano and piRNApredictor**

**Species**	**Method**	**Dataset size**		**t-value**	***Se***	***Sp***	**ACC**
		**Positive**	**Negative**				
*H. sapiens*	piRNApredictor	7,140	2,898	0	97.97%	8.20%	71.48%
	Piano			-	93.67%	44.72%	79.54%
*M. musculus*	piRNApredictor	14,495	2,564	0	83.09%	9.52%	72.03%
	Piano			-	89.10%	44.15%	82.34%
*R. norvegicus*	piRNApredictor	14,195	2,588	0	69.19%	8.42%	59.82%
	Piano			-	89.65%	34.58%	81.16%

**Figure 4:**
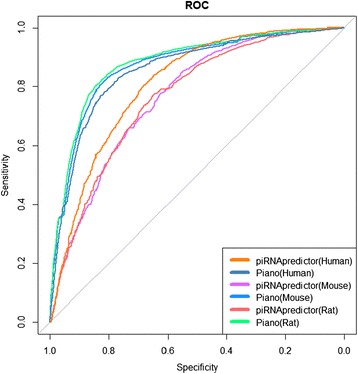
**The ROC curves of the two algorithms.**

As shown in Table 3, using the same datasets for the three species, Piano has prediction specificity of ~40%, which is much higher than that of piRNApredictor (~10%). Figure 4 shows the ROC curves (AUC) of piRNApredictor and Piano. AUC is a global performance measure because it integrates overall threshold values [33]. Clearly, Piano achieves better performance than piRNApredictor in identifying piRNAs.

### Prediction of *Chilo suppressalis* piRNAs

**Figure 5:**
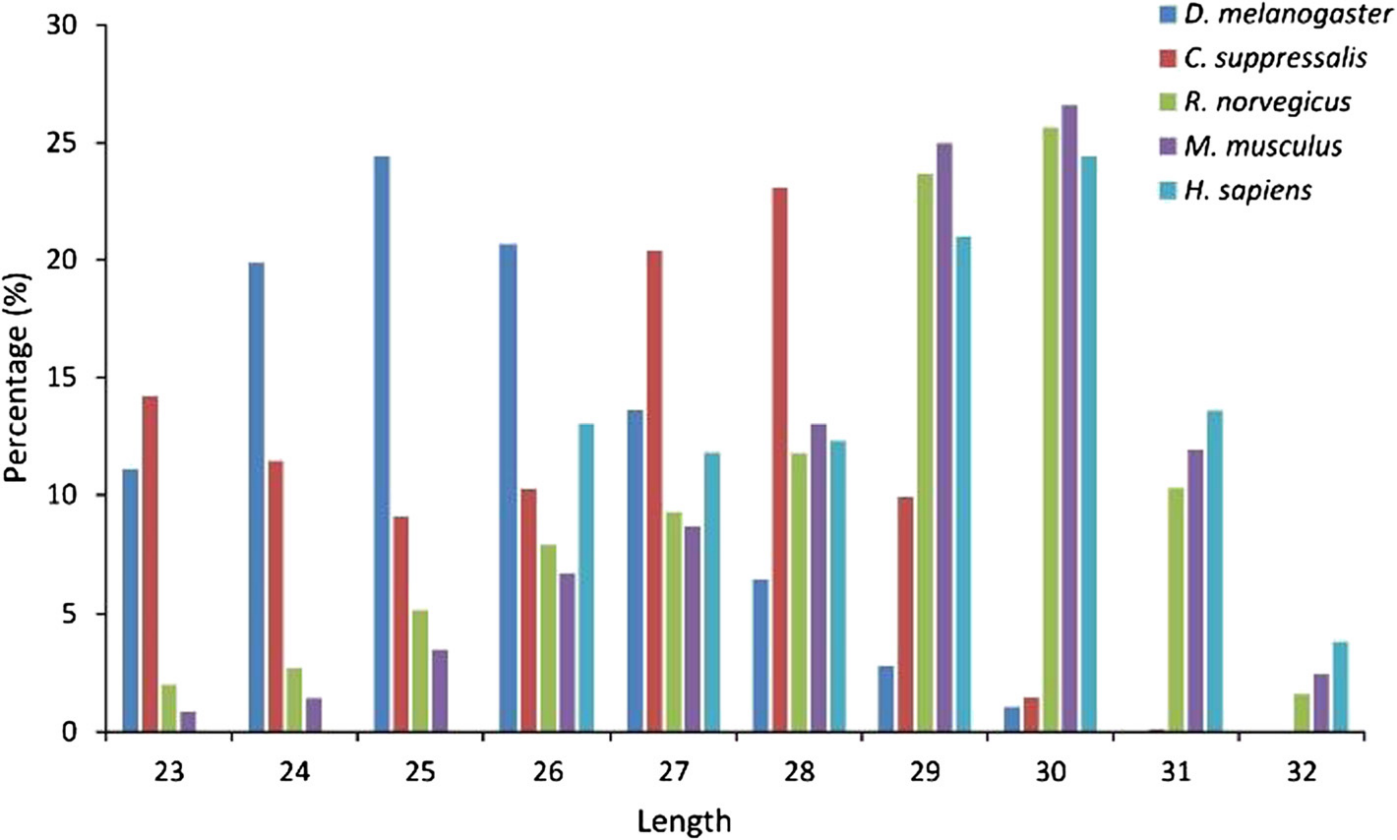
**The length distribution of piRNAs in five species (** ***D. melanogaster, C. suppressalis*** **,** ***R. norvegicus*** **,** ***M. musculus*** **, and** ***H. sapiens*** **).**

Rice striped stem borer (SSB) is an important rice pest that causes huge yield loss. To date, no piRNAs have been reported in SSB. We applied our program to predict piRNAs from small RNA-Seq data; 2,170,655 short sequences in total. From this data, 82,639 piRNAs were predicted. The whole prediction procedure takes ~7 hours on an Ubuntu server (Sugon X8DT6, 2 CPU processors, each has 12 threads, 48 G memory). An interesting discovery is that insect piRNAs might have a different length distribution than mammalian piRNAs. The mammalian piRNAs have a length peak at 29–30 nt, whereas that in *Drosophila* is 24–26 nt and that in SSB is 27–28 nt (Figure 5). These findings are consistent with previous results [14].

### piRNA target sequences

**Figure 6:**
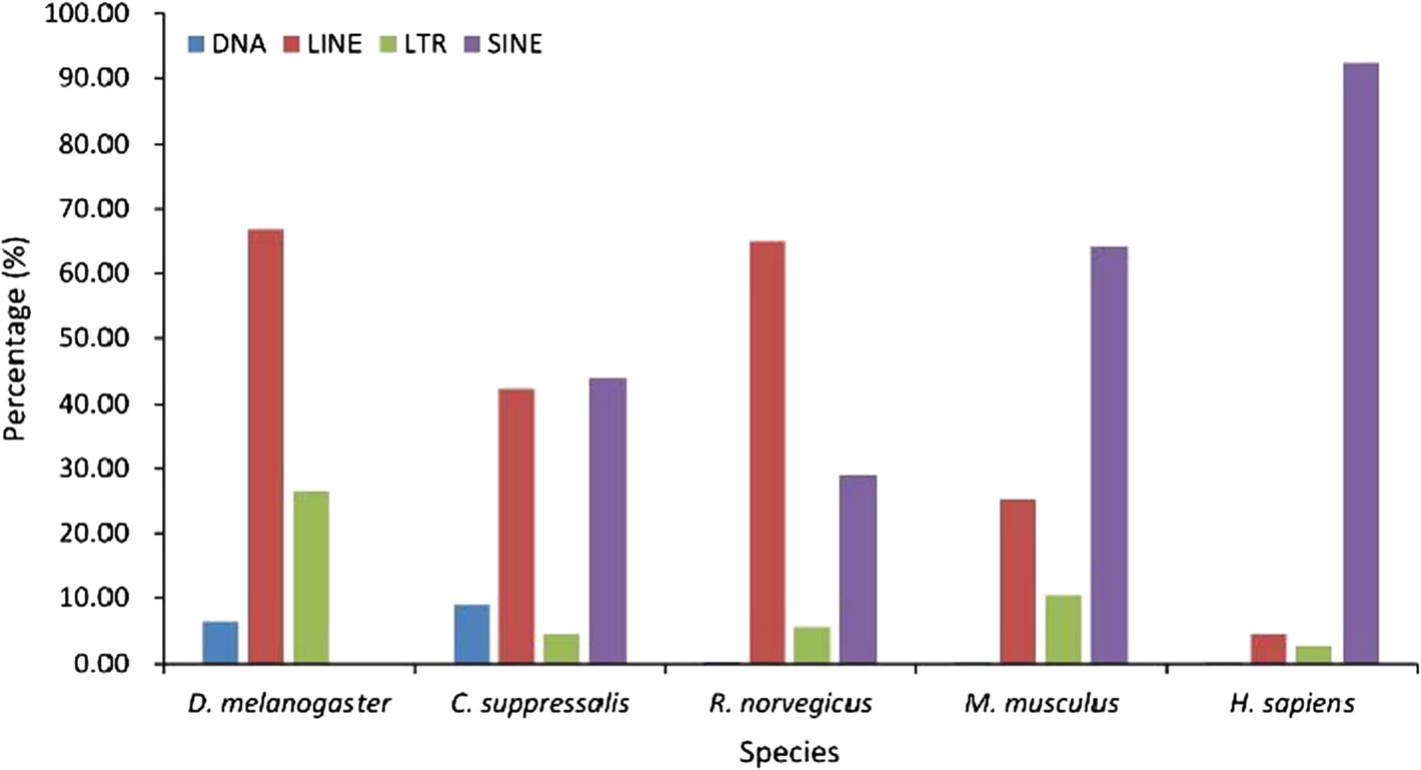
**Percentages of piRNAs paired with different kinds of transposon in five species (** ***D. melanogaster*** **,** ***C. suppressalis*** **,** ***R. norvegicus*** **,** ***M. musculus*** **, and** ***H. sapiens*** **).**

The main function of piRNAs is to target and silence transposons. In this study, we analyzed piRNAs and their target sequences in human, rat, mouse, fruit fly and rice stem borer. We calculated the percentage of piRNAs targeting different categories of transposons. Our data analysis indicated that the majority of human piRNAs (95.0%) target SINE transposons. In mouse, 67.5% of piRNAs target SINE and 24.9% target LINE transposons. In rat, 65.6% of piRNAs target SINE and 29.0% target LINE transposons. In *Drosophila*, 66.8% of piRNAs target LINE and 26.4% target LTR transposons. In SSB, 42.4% of piRNAs target LINE and 44.0% target SINE transposons (Figure 6). These results indicate that piRNAs may have somewhat different mechanisms of action in different species [34,35].

## Conclusions

In this study, we developed a novel program for piRNA annotation called Piano. The program uses piRNA-transposon alignment/pairing and piRNA nucleotide content information (i.e., structure-sequence triplet elements) and achieves a high sensitivity, specificity and accuracy of over 90%. To the best of our knowledge, this is the best prediction performance achieved in comparison with other tools, such as piRNApredictor. Piano can be used not only for large-scale piRNA prediction from small RNA sequencing data but also for genome-wide annotation of piRNAs.

## Additional files

Additional file 1: Table S2. 10-cross validation datasets. Additional file 2: Table S1. An excel sheet of *F* values for each triplet element.

## References

[1] Claverie J-M (2005). Fewer genes, more noncoding RNA. Science.

[2] Mattick JS (2005). The functional genomics of noncoding RNA. Science.

[3] Xie C, Yuan J, Li H, Li M, Zhao G, Bu D, Zhu W, Wu W, Chen R, Zhao Y (2014). NONCODEv4: exploring the world of long non-coding RNA genes. Nucleic Acids Res.

[4] Kutter C, Svoboda P (2008). miRNA, siRNA, piRNA. RNA Biol.

[5] Girard A, Sachidanandam R, Hannon GJ, Carmell MA (2006). A germline-specific class of small RNAs binds mammalian Piwi proteins. Nature.

[6] Grivna ST, Beyret E, Wang Z, Lin H (2006). A novel class of small RNAs in mouse spermatogenic cells. Genes Dev.

[7] Houwing S, Kamminga LM, Berezikov E, Cronembold D, Girard A, Van Den Elst H, Filippov DV, Blaser H, Raz E, Moens CB (2007). A role for Piwi and piRNAs in germ cell maintenance and transposon silencing in Zebrafish. Cell.

[8] Kennedy D (2006). Breakthrough of the Year. Science.

[9] Brennecke J, Aravin AA, Stark A, Dus M, Kellis M, Sachidanandam R, Hannon GJ (2007). Discrete small RNA-generating loci as master regulators of transposon activity in *Drosophila*. Cell.

[10] Thomson T, Lin H (2009). The biogenesis and function PIWI proteins and piRNAs: progress and prospect. Annu Rev Cell Dev Biol.

[11] Juliano C, Wang J, Lin H (2011). Uniting germline and stem cells: the function of Piwi proteins and the piRNA pathway in diverse organisms. Annu Rev Genet.

[12] Lukic S, Chen K: **Human piRNAs are under selection in Africans and repress transposable elements.** *Mol Biol Evol* 2011, **28:**3061–3067. 10.1093/molbev/msr141 PMC3199439 21613236

[13] Lau NC, Seto AG, Kim J, Kuramochi-Miyagawa S, Nakano T, Bartel DP, Kingston RE (2006). Characterization of the piRNA complex from rat testes. Science.

[14] Yin H, Lin H (2007). An epigenetic activation role of Piwi and a Piwi-associated piRNA in *Drosophila melanogaster*. Nature.

[15] Altschul SF, Gish W, Miller W, Myers EW, Lipman DJ (1990). Basic local alignment search tool. J Mol Biol.

[16] Bailey TL, Elkan C (1994). Fitting a mixture model by expectation maximization to discover motifs in bipolymers.

[17] Betel D, Sheridan R, Marks DS, Sander C (2007). Computational analysis of mouse piRNA sequence and biogenesis. PLoS Comput Biol.

[18] Zhang Y, Wang X, Kang L (2011). A k-mer scheme to predict piRNAs and characterize locust piRNAs. Bioinformatics.

[19] Wu J, Liu Q, Wang X, Zheng J, Wang T, You M, Sun ZS, Shi Q (2013). mirTools 2.0 for non-coding RNA discovery, profiling, and functional annotation based on high-throughput sequencing. RNA Biol.

[20] Giurato G, De Filippo MR, Rinaldi A, Hashim A, Nassa G, Ravo M, Rizzo F, Tarallo R, Weisz A (2013). iMir: An integrated pipeline for high-throughput analysis of small non-coding RNA data obtained by smallRNA-Seq. BMC bioinformatics.

[21] Menor M, Baek K, Poisson G (2013). Multiclass relevance units machine: benchmark evaluation and application to small ncRNA discovery. BMC Genomics.

[22] Rosenkranz D, Zischler H (2012). proTRAC-a software for probabilistic piRNA cluster detection, visualization and analysis. BMC bioinformatics.

[23] Jung I, Park JC, Kim S: **piClust: a density based piRNA clustering algorithm.** *Comput Biol Chem* 2014, **50:**60–67. 10.1016/j.compbiolchem.2014.01.008 24656595

[24] Nishida KM, Saito K, Mori T, Kawamura Y, Nagami-Okada T, Inagaki S, Siomi H, Siomi MC (2007). Gene silencing mechanisms mediated by Aubergine–piRNA complexes in *Drosophila* male gonad. RNA.

[25] Jiang H, Wong WH (2008). SeqMap: mapping massive amount of oligonucleotides to the genome. Bioinformatics.

[26] Karolchik D, Barber GP, Casper J, Clawson H, Cline MS, Diekhans M, Dreszer TR, Fujita PA, Guruvadoo L, Haeussler M (2014). The UCSC genome browser database: 2014 update. Nucleic Acids Res.

[27] Bu D, Yu K, Sun S, Xie C, Skogerbo G, Miao R, Xiao H, Liao Q, Luo H, Zhao G Zhao H, Liu Z, Liu C, Chen R, Zhao Y: **NONCODE v3. 0: integrative annotation of long noncoding RNAs.** *Nucleic Acids Res* 2012, **40:**D210–215. 10.1093/nar/gkr1175 PMC3245065 22135294

[28] Tafer H, Hofacker IL (2008). RNAplex: a fast tool for RNA–RNA interaction search. Bioinformatics.

[29] Chang C-C, Lin C-J (2011). LIBSVM: a library for support vector machines. ACM Transactions on Intelligent Systems and Technology (TIST).

[30] Xue C, Li F, He T, Liu G-P, Li Y, Zhang X (2005). Classification of real and pseudo microRNA precursors using local structure-sequence features and support vector machine. BMC bioinformatics.

[31] Dror G, Sorek R, Shamir R (2005). Accurate identification of alternatively spliced exons using support vector machine. Bioinformatics.

[32] Golub TR, Slonim DK, Tamayo P, Huard C, Gaasenbeek M, Mesirov JP, Coller H, Loh ML, Downing JR, Caligiuri MA (1999). Molecular classification of cancer: class discovery and class prediction by gene expression monitoring. Science.

[33] Agarwal S, Graepel T, Herbrich R, Har-Peled S, Roth D (2005). Generalization bounds for the area under the ROC curve. Journal of Machine Learning Research.

[34] Huang CR, Burns KH, Boeke JD (2012). Active transposition in genomes. Annu Rev Genet.

[35] Smit AF (1999). Interspersed repeats and other mementos of transposable elements in mammalian genomes. Curr Opin Genet Dev.

